# Characteristics of diabetic macular edema patients refractory to anti-VEGF treatments and a dexamethasone implant

**DOI:** 10.1371/journal.pone.0222364

**Published:** 2019-09-12

**Authors:** Moon Young Choi, Donghyun Jee, Jin-woo Kwon

**Affiliations:** Department of Ophthalmology and Visual Science, St. Vincent’s Hospital, College of Medicine, The Catholic University of Korea, Suwon, Korea; Massachusetts Eye & Ear Infirmary, Harvard Medical School, UNITED STATES

## Abstract

**Purpose:**

To determine the characteristics of diabetic macular edema (DME) patients refractory to intravitreal bevacizumab (IVB) treatments and an additional dexamethasone implant.

**Methods:**

We classified 119 DME patients according to whether or not they are responsive to 3 consecutive monthly anti-VEGF treatments and/or an additional dexamethasone implant. We compared their concentrations of IL (interleukin)-1β, IL-8, IL-10, IL-17, placental growth factor (PlGF), and vascular endothelial growth factor (VEGF) in the aqueous humor as well as their optical coherence tomography (OCT) findings, and baseline characteristics. We used logistic regression analyses to identify preoperative factors related to refractoriness to treatments.

**Results:**

Of 119 treatment-naïve DME patients, 50 (42.02%) patients showed responsiveness [central subfield thickness (CST) < 300μm] after 3 IVBs, and 59 (49.58%) patients showed responsiveness after an additional dexamethasone implant, but 10 (8.40%) patients showed CST 300 ≥ μm even after both treatments. Refractory DME patients showed significantly higher number of hyperreflective foci (HF) in the OCT and higher average level of aqueous IL-1β at baseline (p<0.001 and p = 0.042, respectively). In the logistic regression analysis, higher number of HF in the OCT was associated with the refractoriness to both treatments (odds ratio [OR]: 7.03, p = 0.007)

**Conclusions:**

Higher number of HF in the OCT at the initial visit was associated with poor responses to IVBs and an additional dexamethasone implant.

## Introduction

Diabetic retinopathy (DR) is one of the most significant causes of visual impairment worldwide.[1] A common cause of visual disturbance in DR is diabetic macular edema (DME),[1–3] characterized by damage, in the early phase, to the inner blood–retina barrier caused by metabolic changes and inflammation.[4, 5] The inflammation is involved by inflammatory cells, cytokines, growth factors, and enzymes.[5, 6] In the past, laser treatment and vitrectomy were commonly used to treat DME.[7–9] Recently, given studies revealing the fundamental role played by vascular endothelial growth factor (VEGF) [10], anti-VEGF antibodies have become the preferred treatment.[11] In addition, the availability of micronized dexamethasone in a biodegradable copolymer allows the steroid to be easily used to counter the inflammation that plays a role in DME pathogenesis. Dexamethasone is effective at reducing central subfield thickness (CST) and improving visual acuity in DME patients.[12, 13]

Although several treatment options are available, no consensus DME treatment based on patient status has yet been achieved. Several studies have sought to predict prognosis or responsiveness to various treatment options, using optical coherence tomography (OCT), measurements of biomarkers in the ocular fluid, or systemic evaluation.[14–17] In this study, we measured levels of IL-1β, IL-8, IL-10, IL-17, placental growth factor (PlGF), and VEGF in aqueous humor; systemic factors including duration of diabetes and glycated hemoglobin levels (HbA1c); and ocular parameters of 119 treatment-naive DME patients in terms of their responsiveness to intravitreal bevacizumab (IVB) and an additional intravitreal dexamethasone implant.

## Methods

We followed all relevant tenets of the Declaration of Helsinki. This was a prospective study and protocol was approved by the institutional review/ethics board of the Catholic University of Korea. All participants gave written informed consent for the use of their clinical records.

We enrolled treatment-naive DME eyes of type II DM patients with CST ≥300 μm.[18] The exclusion criteria included glaucoma, retinal degeneration, and macular edema attributable to other causes, including an epiretinal membrane or vitreo-macular traction. We excluded eyes with concurrent diseases such as retinal vascular occlusion and eyes with histories of prior ocular conditions, uveitis, or intraocular therapy and laser that could influence enzyme levels in the aqueous humor.

When patients were diagnosed with DME, we measured their HbA1c levels, and all patients underwent full ophthalmological examinations, including measurement of best corrected visual acuity (BCVA) and dilated fundus examination. All eyes were classified using the Early Treatment of Diabetic Retinopathy criteria as having mild non-proliferative diabetic retinopathy (NPDR), moderate NPDR, severe NPDR, or proliferative diabetic retinopathy (PDR). Macular thickness was measured via optical coherence tomography (OCT; Cirrus High-Definition OCT; Carl Zeiss Meditec, Dublin, CA, USA). The hyperreflective foci (HF) were manually measured to within 1,500 μm, and ellipsoid zone (EZ) disruption was manually measured to within 1,000 μm using a horizontal scan centered on the fovea. [19] The EZ disruption was graded as 0 when intact, 1 in cases of focal disruption ≤ 200 μm in length, and 2 in cases of disruption > 200 μm in length. [20]

**Fig 1:**
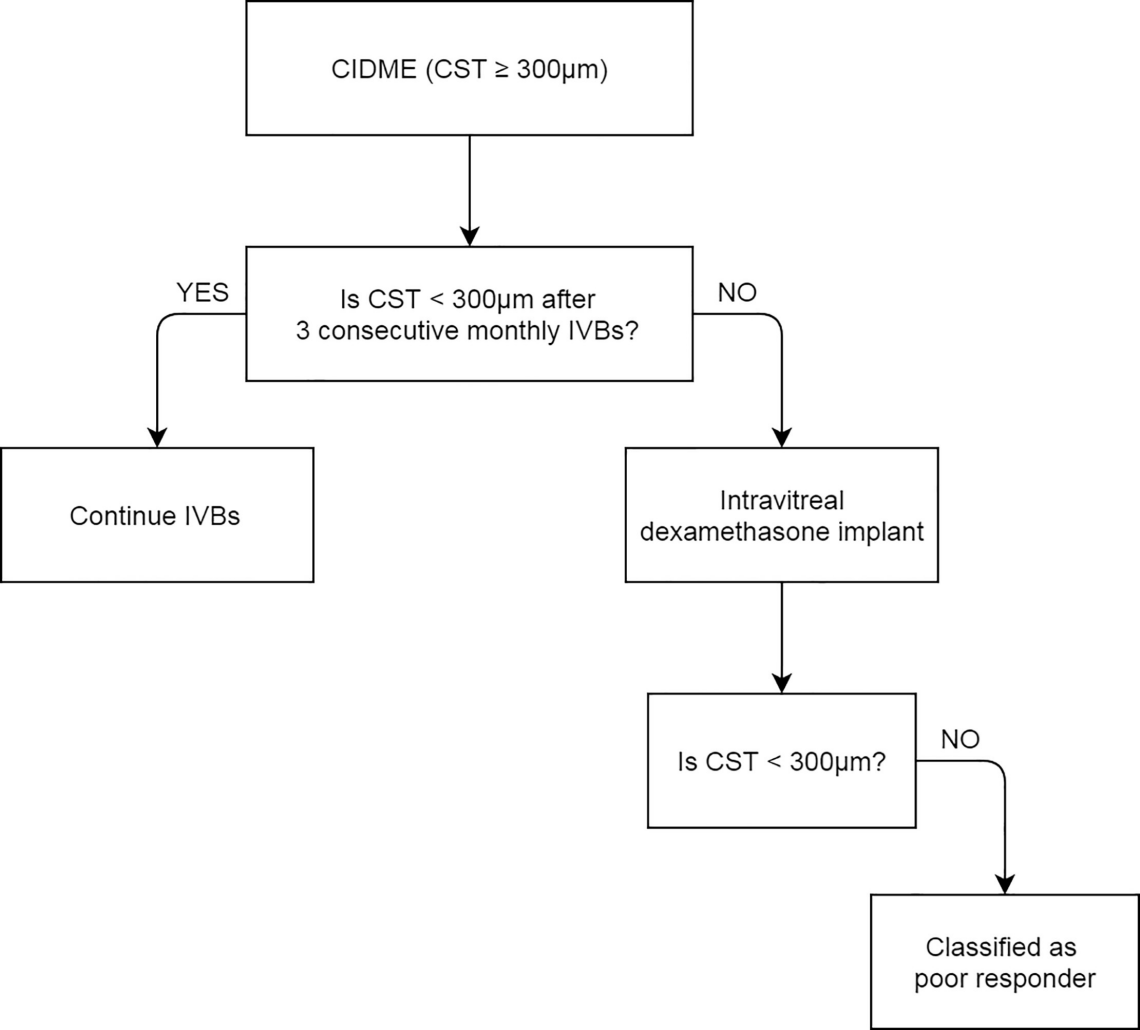
Flowchart showing treatment decision tree for CIDME. Naïve CIDME patients were treated with 3 consecutive IVBs and if CST after treatments was reduced to < 300μm then IVB was continued. If their CST ≥ 300μm after 3 consecutive IVBs, intravitreal dexamethasone implant was inserted. If these patients still remained to show CST > 300μm, then they were classified as poor responders. CIDME = Center involving diabetic macular edema; CST = Central subfield thickness; IVB = intravitreal bevacizumab.

We classified DME patients as either good or poor responders. Responsiveness was defined as CST < 300 μm after treatment. We first administered three consecutive monthly injections of with 1.25mg dose of IVB and evaluated the CST 1 month after the third injection. In poor responders, we additionally placed a dexamethasone implant (Ozurdex^®^; Allergan Inc., Irvine, CA, USA), and we evaluated these patients whether or not achieving CST <300 μm within 3 months after treatment. Responsive group was defined as patients who achieved CST < 300μm after 3IVBs with or without dexamethasone implant and refractory group was defined as patients who showed CST ≥ 300μm even after both treatments (Fig 1). [21, 22] We followed all the patients until 6month after initial treatments.

## Assays of cytokines and growth factors

Concentrations of IL-1β, IL-8, IL-10, IL-17, PlGF, and VEGF in 75 μL amounts of aqueous humor from the anterior chamber (collected via anterior paracentesis at the first IVB injection immediately after diagnosed with CIDME) were measured using bead-immobilized human antibodies against IL-1β, IL-8, IL-10, IL-17, PlGF, and VEGF. The humor samples were mixed with 75 μL amounts of Calibrator Diluent RD6-52 and added to the bead preparations. Then we incubated the samples for 2 h at room temperature (20–25°C), for a further 1 h at room temperature after the addition of detector antibodies, and for 30 min at room temperature after the addition of the streptavidin-phycoerythrin reagent. A Luminex-x-MAP suspension array system (Luminex, Austin, TX, USA) was used for detection; this is a multiplexed, microsphere suspension immunoassay that detects and quantitates spectrally unique microspheres attached to specific antibodies. The technique enables many samples to be analyzed in a single reaction. The detection limits and dynamic ranges are as follows: 0.8 pg/mL with a dynamic range to 3,950 pg/mL for IL-1β, 1.8 pg/mL with a dynamic range to 1,140 pg/mL for IL-8, 1.6 pg/mL with a dynamic range to 890 pg/mL for IL-10, 1.8 pg/mL with a dynamic range to 2,090 pg/mL for IL-17, 1.9 pg/mL with a dynamic range to 470 pg/mL for PlGF, and 2.1 pg/mL with a dynamic range to 2,170 pg/mL for VEGF. All values under the lower limit of detection were considered zero values.

## Statistical evaluation

All statistical analyses were performed using SPSS for Windows (ver. 21.0; SPSS, Chicago, IL, USA) and R (ver. 3.2.3, 2015-12-10, Platform: x86_64-redhat-linux-gnu, R Core Team (2015) [R: A language and environment for statistical computing. R Foundation for Statistical Computing, Vienna, Austria. URL https://www.R-project.org/]).

The t-test, Mann-Whitney U-test, and chi-square test were used to compare the values or the ratios of the patient groups. Logistic regression analyses were used to identify factors affecting responsiveness to IVB and/or the dexamethasone implant. The level of statistical significance was set at p<0.05.

## Results

We enrolled 119 treatment-naïve CIDME eyes of 119 patients. The mean age was 57.59 ± 9.17 years, and there were 51 males and 68 females. In terms of DR staging, 71 (59.66%) patients had proliferative DR and 48 (40.34%) patients had non-proliferative DR. The mean BCVA (LogMAR) was 0.56 ± 0.34, and the mean CST was 397.92 ± 83.14 μm at baseline. When classifying the DME morphology as cystoid macular edema (CME) or diffuse retinal thickening (DRT), 42 (35.29%) patients had CME and 77 (64.71%) had DRT. Of 119 DME patients, 50 (42.02%) patients showed CST < 300μm after 3 IVBs, and 59 (49.58%) patients showed CST < 300μm after an additional dexamethasone implant, ten (8.40%) patients showed CST 300 ≥ μm after both treatments. The systemic and ocular characteristics of responsive to IVB and/or dexamethasone treatments group and refractory to both treatments group are summarized in Table 1. There was a significant difference in HF in OCT between two groups (p<0.001). When comparing cytokine levels in the aqueous humor, the aqueous IL-1β level of refractory DME group was higher compared to that of responsive DME group (p = 0.042).

**Table 1:** Demographics and clinical characteristics of DME patients classified with responsiveness.

			Responsive DME (n = 109)	Refractory DME (n = 10)	p
Systemic factors	Sex (male:female)		64:45	4:6	0.418
	Age (years)		57.00 [53.00;64.00]	60.00 [57.00;63.00]	0.396
	Duration of diabetes		10.00 [3.00;17.00]	7.00 [3.00;16.00]	0.715
	HbA1C (%)		7.61 ± 1.00	7.06 ± 1.25	0.105
OCT findings	Number of HF		5.0 [3.0;9.0]	14.0 [9.0;19.0]	<0.001
	DME type (DRT:CME)		72:37	5:5	0.502
	EZ disruption grade	0	72 (66.06%)	5 (50.00%)	0.341
		1	29 (26.61%)	3 (30.00%)	
		2	8 (7.34%)	2 (20.00%)	
Aqueous humor	IL-1β (pg/mL)		0.00 [0.00;3.49]	2.71 [0.00;12.73]	0.042
	IL-8 (pg/mL)		15.05 [9.40;22.64]	10.75 [3.49;17.47]	0.094
	IL-10 (pg/mL)		0.00 [0.00;0.00]	0.00 [0.00;0.00]	0.195
	IL-17 (pg/mL)		0.00 [0.00;2.56]	1.28 [0.00;3.37]	0.306
	PlGF (pg/mL)		2.41 [0.00;4.04]	2.19 [0.00;2.85]	0.585
	VEGF (pg/mL)		62.37 [27.64;102.10]	77.74 [63.57;84.27]	0.578
Ocular factors	Baseline BCVA (LogMAR)		0.50 [0.30;0.70]	0.60 [0.30;1.00]	0.467
	Baseline CST (μm)		371.0 [340.0;412.0]	406.50 [351.0;592.0]	0.160
	DMR stage	Moderate NPDR	18 (16.51%)	2 (20.00%)	0.574
		Severe NPDR	27 (24.77%)	1 (10.00%)	
		PDR	64 (58.72%)	7 (70.00%)	

Values are expressed as mean ± SD or median and interquartile range, as appropriate.

DME, diabetic macular edema; HbA1c, glycated hemoglobin; HF, hyperreflective foci; CME, cystoid macular edema, DRT, diffuse retinal thickening; EZ, ellipsoid zone; IL, interleukin; PlGF, placental growth factor; VEGF, vascular endothelial growth factor; BCVA, best-corrected visual acuity; CST, central subfield thickness; DMR, DM retinopathy; NPDR, non-proliferative diabetic retinopathy; PDR, proliferative diabetic retinopathy

The factors identified as associated with refractoriness are summarized in Table 2. In multivariate logistic analyses, higher number of HF (>10) in the OCT was associated with the refractoriness to both treatments (odds ratio [OR]: 7.03, p = 0.007)

**Table 2:** Results of logistic regression of the effects of responsiveness to treatments.

	Category	n(%)	Univariate		Multivariate	
			OR (95%CI)	p	OR (95%CI)	p
Sex	Female	68 (57.14%)	Reference			
	Male	51 (42.86%)	2.13 (0.58, 8.75)	0.261		
Age (years)	≤ 60	70 (58.82%)	Reference			
	> 60	49 (41.18%)	1.48 (0.39, 5.60)	0.556		
DMR stage	NPDR	48 (40.34%)	Reference			
	PDR	71 (59.66%)	1.64 (0.43, 7.93)	0.490		
EZ disruption	(-)	77 (64.71%)	Reference			
	(+)	42 (35.29%)	1.95 (0.51, 7.41)	0.316		
Number of HF	≤ 10	97 (81.51%)	Reference		Reference	
	> 10	22 (18.49%)	8.72 (2.25, 37.50)	0.002	7.03 (1.72, 31.76)	0.007
Aqueous IL-1β (pg/mL)	≤ 0.80	61 (51.26%)	Reference		Reference	
	> 0.80	58 (48.74%)	2.65 (0.70,12.81)	0.173	1.66 (0.38, 8.59)	0.511
Aqueous IL-8 (pg/mL)	≤ 14.89	60 (50.42%)	Reference			
	> 14.89	59 (49.58%)	0.41 (0.08, 1.54)	0.208		
Aqueous IL-10 (pg/mL)	≤ 1.60	70 (58.82%)	Reference			
	> 1.60	49 (41.18%)	1.48 (0.39, 5.60)	0.556		
Aqueous IL-17 (pg/mL)	≤ 1.80	60 (50.42%)	Reference			
	> 1.80	59 (49.58%)	2.22 (0.60, 9.10)	0.238		
Aqueous VEGF (pg/mL)	≤ 64.23	60 (50.42%)	Reference		Reference	
	> 64.23	59 (49.58%)	2.56 (0.67, 12.35)	0.190	2.01 (0.48, 10.22)	0.355
Aqueous PlGF (pg/mL)	≤ 2.41	65 (54.62%)	Reference			
	> 2.41	54 (45.38%)	0.79 (0.19, 2.91)	0.722		

OR, odds ratio; CI, confidence interval; DMR, DM retinopathy; EZ, ellipsoid zone; HF, hyperreflective foci; IL, interleukin; VEGF, vascular endothelial growth factor; PlGF, placental growth factor

## Discussion

The pathogenesis of DME is complex; the ischemia and the inflammation are closely connected.[5, 23] Several treatment options are now available. Photocoagulation of the point of leakage using laser treatment is used to treat noncenter- involved DME.[24] Removal of tractional components, increasing the clearance rates of inflammatory cytokines and VEGF, increasing the oxygen level of the vitreous via vitrectomy have all been successfully used to treat refractory DME.[9, 25, 26] However, currently the principal treatment is intravitreal injection of anti-VEGF antibodies or steroids, which are both effective and convenient.[11–13] However, the responsiveness of DME patients to treatments differs because they vary in systemic status and/or ocular factors. Long-lasting chronic DME can compromise visual acuity; early optimal treatment is required to reduce CST and establish a normal macular contour. In the absence of such treatment, permanent visual disturbance may develop.[27, 28] Thus, it is necessary to identify factors associated with responsiveness by patient status and to customize treatment. In the present study, we explored associations between responsiveness to treatments with levels of aqueous biomarkers and ocular and systemic factors.

In this study, we found that the aqueous IL-1β level and the initial HF in the OCT were higher in the patients who showed refractoriness to both IVB and the additional dexamethasone implant. And higher number of HF (>10) was associated with this refractoriness in the multivariate logistic regression analysis.

IL-1β has key roles in the inflammatory process such as induction of pro-inflammatory proteins, differentiation or development of inflammatory cells [29]. There are some studies that showed IL-1β is involving diabetic complications associated with inflammatory conditions in type 2 DM [30, 31]. One article suggested that IL-1β accelerates apoptosis of retinal capillary cells, which play part in development of diabetic retinopathy [32]. But another study showed systemic IL-1β inhibition did not affect neovascularization in DR [33].

Previously, HF on OCT were described as features of lipoprotein extravasation in patients with DME [34]. However, recent studies have suggested that they are activated form of microglia and are involved in inflammation [35, 36]. Some following studies have reported that more HF on OCT could be a finding for poor prognosis after anti-VEGF treatments [37, 38]. Our results newly suggested that HF may be indicative of poor responsiveness of DME not only in anti-VEGF but also in steroid implant treatment.

The role of HbA1c in DME remains controversial.[17, 39–41] Some earlier studies found that the extent of DME was associated with the HbA1c level,[40, 41] but more recent studies have found no such association.[17, 39, 42] Here we found that responsiveness to treatments was not associated with the HbA1c level. The group that responded poorly to both agents had lower HbA1c levels than the other group, but the difference was not significant.

Our study has certain limitations. First, we did not use OCT angiography, or fluorescein angiography to evaluate patients. Second, our sample size was relatively small and the follow-up period was short. DME treatment extends over 2 years, and the final visual outcome and CST should be evaluated long term.[43] Third, changes in the levels of aqueous biomarkers after consecutive IVB and dexamethasone treatment would aid in the evaluation of responses to these agents[44], but we lacked such data.

In summary, higher number of HF in the OCT were associated with refractoriness to DME treatments. Additional studies with greater number of participants are required to confirm our results and to elucidate the pathogenesis of DME further.
